# Supplementary material for: Distinct Pathological Changes in Preweaning Mice Infected with Live-Attenuated Rift Valley Fever Virus Strains
Source: Viruses. 2024 Jun 21;16(7):999. doi: 10.3390/v16070999 (PMC11281583; doi:10.3390/v16070999)
Supplement: Supplementary file 1 [file viruses-16-00999-s001.zip › viruses-3060641-supplementary.pdf]

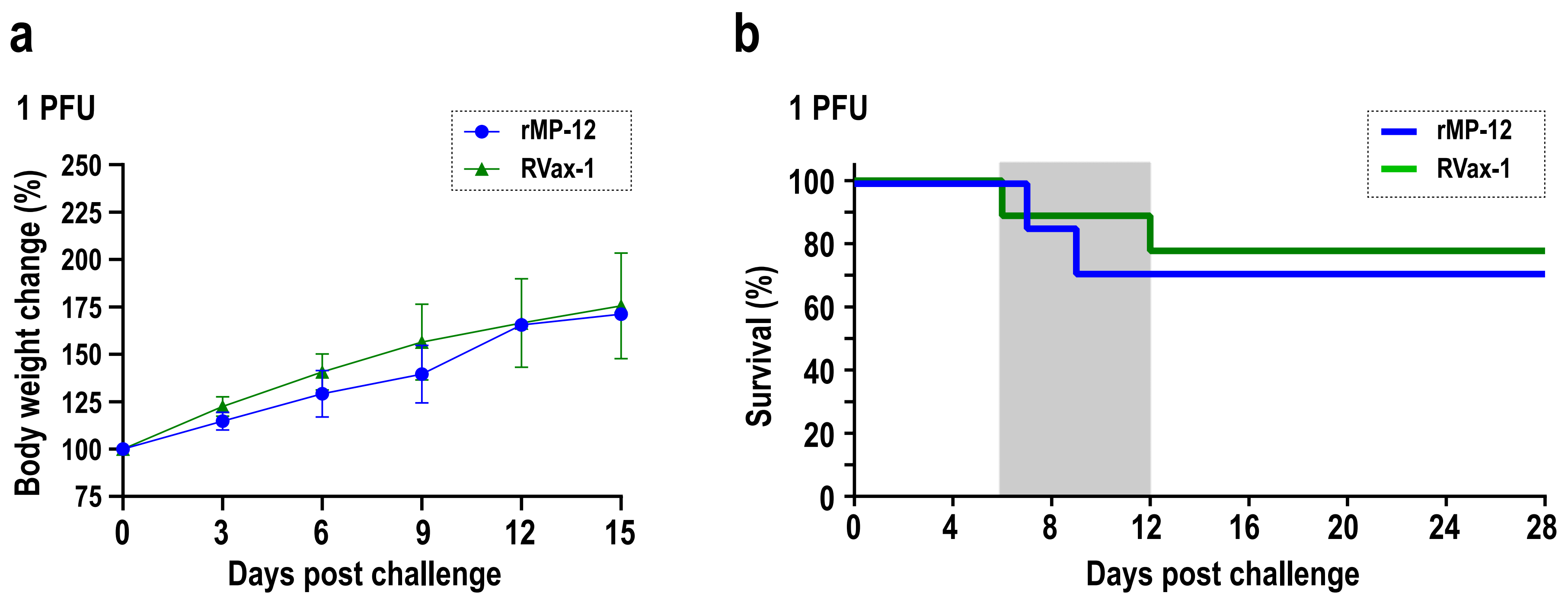

Supplementary Figure S1. The susceptibility of preweaning mice to Rift Valley fever virus MP-12 or RVax-1 at 1 PFU dose. The graph displays changes in body weight (%) (a) and Kaplan–Meier survival curves (b) of 19-day-old C57BL/6 mice challenged intraperitoneally (i.p.) with 1 PFU dose of rMP-12 (blue) or RVax-1 (green). Mice exhibiting over 20% body weight loss and/or severe clinical symptoms were euthanized, while surviving mice were euthanized at 28 days post-challenge (dpc). Error bars represent the standard deviations.
